# LLL12B, a Novel Small-Molecule STAT3 Inhibitor, Induces Apoptosis and Suppresses Cell Migration and Tumor Growth in Triple-Negative Breast Cancer Cells

**DOI:** 10.3390/biomedicines10082003

**Published:** 2022-08-18

**Authors:** Li Pan, Xiang Chen, Feyruz Virgilia Rassool, Chenglong Li, Jiayuh Lin

**Affiliations:** 1 Department of Biochemistry and Molecular Biology, School of Medicine, University of Maryland, Baltimore, MD 21201, USA; 2 Department of Radiation Oncology, School of Medicine, University of Maryland, Baltimore, MD 21201, USA; 3 Department of Medicinal Chemistry, College of Pharmacy, University of Florida, Gainesville, FL 32610, USA

**Keywords:** STAT3, small-molecule inhibitor, triple-negative breast cancer, IL-6 signaling pathway, apoptosis, cell migration, tumor growth

## Abstract

Persistent STAT3 signaling plays a pivotal role in human tumor malignancy, including triple-negative breast cancer (TNBC). There are few treatment options currently available for TNBC; thus, given its importance to cancer, STAT3 is a potential cancer therapeutic target and is the focus of drug discovery efforts. In this study, we tested a novel orally bioavailable small-molecule STAT3 inhibitor, LLL12B, in human MDA-MB-231, SUM159, and murine 4T1 TNBC cell lines. TNBC cells frequently expressed persistent STAT3 phosphorylation and their cell viability was sensitive to STAT3 knockdown by siRNA. LLL12B selectively inhibited the IL-6-mediated induction of STAT3 phosphorylation, but had little effect on the IFN-γ-mediated induction of STAT1 phosphorylation nor the EGF-mediated induction of ERK phosphorylation. In addition, targeting STAT3 with LLL12B induced apoptosis, reduced colony formation ability, and inhibited cell migration in TNBC cells. Furthermore, LLL12B suppressed the tumor growth of the MDA-MB-231 TNBC cells in a mammary fat pad mouse tumor model in vivo. Together, our findings support the concept that targeting persistent STAT3 signaling using the novel small-molecule LLL12B is a potential approach for TNBC therapy.

## 1. Introduction

Breast cancer is a heterogeneous disease and has become the most diagnosed cancer in women worldwide [1]. It is composed of different subtypes [2], one such subtype being triple-negative breast cancer (TNBC), where tumors lack the expression of estrogen receptor-alpha (ERα), progesterone receptor (PR), and HER2 [3]. TNBC accounts for approximately 15–20% of all breast cancers, and unlike other subtypes of breast cancer, TNBC is more aggressive, has a greater distant metastasis potential, and a poorer survival rate [4].

Due to TNBC’s genomic heterogeneity and lack of validated biomarkers, TNBC treatment is very challenging. The main treatment for early-stage TNBC is chemotherapy followed by surgery. Recently, poly-ADP-ribose polymerase (PARP) inhibitors were approved for the treatment of metastatic breast cancers with germline BRCA mutations [5]. Immune checkpoint inhibitors (ICIs) targeting programmed cell death protein 1 (PD-1)/programmed cell death-ligand 1 (PD-L1), such as pembrolizumab, in combination with chemotherapy, were also approved for patients with metastatic TNBC expressing PD-L1 [6,7]. Despite the encouraging results of PARP inhibitors and immunotherapy for TNBC, their use remains limited for patients with germline BRCA mutations and PD-L1 expression. Therefore, developing novel therapeutic targets is crucial for TNBC treatment.

Recent advances have been made in understanding the molecular heterogeneity of TNBC and several potential therapeutic targets such as PI3K/AKT/mTOR (phosphatidylinositol 3-kinase/AKT/mammalian target of the rapamycin), CDKs (cyclin-dependent kinases), EGFR (epidermal growth factor receptor), VEGFR (vascular endothelial growth factor receptor), AR (androgen receptor), and STAT3 (signal transducer and activator of transcription 3) have been explored [8,9,10,11]. Notably, STAT3 is persistently activated in various cancers, including breast cancer, and is often activated and required for the growth and survival of TNBC cells [12,13,14].

Accumulating evidence shows that persistent STAT3 signaling is crucial for cell proliferation [15], cell invasion and migration [16], chemoresistance [17], angiogenesis, immune regulation [18], cell metabolism [19], and the maintenance of stem cell properties in TNBC [11,14]. In addition, TNBC patients with the elevated expression of p-STAT3 have been associated with a decreased probability of relapse-free survival compared to those with a low expression of p-STAT3 [20,21]. Therefore, targeting STAT3 is likely to be a potential strategy for TNBC therapy. A variety of STAT3 inhibitors with different mechanisms have been developed and a few of them are in clinical trials, but there are no FDA-approved STAT3 inhibitors for treatment to date [22]. The development of specific and effective novel STAT3 inhibitors for potential cancer prevention and therapy is desirable. With advances in computer-aided drug design, we were able to develop several selective STAT3 inhibitors using the advanced multiple ligand simultaneous docking method (AMLSD) [23,24,25]. We identified that LLL12B, a prodrug of LLL12, is activated by the tumor-associated plasmin through the hydrolytic cleavage of the carbamate ester bond to release LLL12 [26]. It directly binds to the pTyr705 binding site of STAT3 with improved in vivo pharmacokinetic properties relative to the parent drug LLL12, which supports that it has superior and selective inhibition of STAT3 [25,27,28]. In this study, we tested LLL12B in TNBC cells and the results showed that targeting STAT3 with LLL12B induced apoptosis and suppressed colony formation, migration, and tumor growth in TNBC cells. Taken together, our findings offer the orally bioavailable STAT3 inhibitor, LLL12B, as a novel therapeutic agent for potential TNBC therapy.

## 2. Materials and Methods

### 2.1. Cell Culture and Reagents

Human breast cancer cell lines T47D, MDA-MB-231, SUM159, and the murine TNBC cell line 4T1 were used in this study. The cells were cultured in Dulbecco’s Modified Eagle Medium (Corning, New York, NY, USA) supplemented with 10% fetal bovine serum (Sigma-Aldrich, St. Louis, MO, USA) and 1% Penicillin/Streptomycin (Sigma-Aldrich, St. Louis, MO, USA). All the cells were maintained at 37 °C in a humidified atmosphere with 5% CO_2_.

LLL12B was synthesized by the laboratory of Dr. Chenglong Li at the University of Florida College of Pharmacy and C188-9 was purchased from MedChemExpress LLC (Monmouth Junction, NJ, USA). LLL12B and C188-9 were dissolved in sterile dimethyl sulfoxide (DMSO). Human Interleukin-6 (IL-6), Interferon-γ (IFN-γ), and Epidermal Growth Factor (EGF) were prepared according to the manufacturer’s instructions (Cell Signaling Technology, Danvers, MA, USA).

### 2.2. SiRNA and Transfection

STAT3 siRNA and Negative Control siRNA (Cell Signaling Technology, Danvers, MA, USA) were used to knockdown STAT3 in the MDA-MB-231, SUM159, and 4T1 cell lines. Cells were transfected with STAT3 siRNA or Negative Control siRNA using Lipofectamine RNAiMAX (Invitrogen, Carlsbad, CA, USA) according to the manufacturer’s instructions. After 72 h, cell viability was examined by the MTT cell viability assay, and the transfection efficiency was examined by Western blot analysis.

### 2.3. MTT Cell Viability Assay

MDA-MB-231, SUM159, and 4T1 cells were plated in a 96-well plate in triplicate. After overnight incubation, the cells were transfected with STAT3 siRNA or Negative Control siRNA for 72 h. Subsequently, 20 µL of 5 mg/mL thiazolyl blue tetrazolium dye solution (Sigma-Aldrich, St. Louis, MO, USA) was added to each well of the plate and incubated at 37 °C for 4 h. One hundred µL of N, N-dimethylformamide solution (Sigma-Aldrich, St. Louis, MO, USA) was used to dissolve the formazan. Absorbance at 595 nm was read using an EL808 Ultra Microplate Reader (BioTek, Winooski, VT, USA).

### 2.4. Western Blot Analysis

Cells were collected and the total amount of protein was extracted using cell lysis buffer (Cell Signaling Technology, Danvers, MA, USA). Protein concentration was determined using the Pierce BCA Protein Assay Kit (Thermo Fisher Scientific, Waltham, MA, USA) according to the manufacturer’s instructions. Equal amounts of protein were separated by 10% SDS–PAGE gels and transferred to PVDF membranes. Membranes were blocked in 5% non-fat milk at room temperature and probed with specific antibodies P-STAT3 (Y705), STAT3, cyclin D1, cleaved caspase-3, P-STAT1 (Y701), STAT1, P-ERK, ERK, or GAPDH (1:1000, Cell Signaling Technology, Danvers, MA, USA) at 4 °C overnight. The blots were visualized using SuperSignal^TM^ West Femto Maximum Sensitivity Substrate (Thermo Fisher Scientific, Waltham, MA, USA) and an Amersham Imager 680 (GE Healthcare Life Sciences, Marlborough, MA, USA) after incubation with horseradish peroxidase (HRP)-conjugated anti-rabbit secondary antibody (1:5000, Cell Signaling Technology, Danvers, MA, USA).

### 2.5. Immunofluorescence Staining

SUM159 cells were seeded in a 6-well plate and treated with LLL12B or DMSO for 8 h. The cells were then fixed with 4% paraformaldehyde (PFA) for 15 min at room temperature and permeabilized with ice-cold 100% methanol for 10 min at –20 °C. After blocking in 3% Bovine Serum Albumin (BSA)/0.1% Triton X-100, cells were incubated with rabbit anti-Phospho-Stat3 (Tyr705) (1:100, Cell Signaling Technology, Danvers, MA, USA) overnight at 4 °C, then with secondary antibody for 1 h at room temperature. The cells were photographed using a Leica fluorescence microscope (Leica Microsystems Inc., Deerfield, IL, USA).

### 2.6. Caspase-3/7 Activity Assay

The Caspase-3/7 Fluorescence Assay Kit (Cayman, Ann Arbor, MI, USA) was used to detect the activation of caspase-3/7 and the assay was performed according to the manufacturer’s instructions. Briefly, MDA-MB-231, SUM159, and 4T1 cells were seeded in a 96-well plate in triplicate. After overnight incubation, the cells were treated with either LLL12B or DMSO for 5 h and then lysed and processed to measure caspase-3/7 activity and the fluorescence intensity was read (excitation = 485 nm; emission = 535 nm).

### 2.7. Flow Cytometry Analysis

Flow cytometry was performed to detect cell apoptosis. The APC Annexin V Apoptosis Detection Kit (Biolegend, San Diego, CA, USA) was used for Annexin V staining. The cells were seeded in a 35 mm culture dish at a density of 1 × 10 ^5^ cells/dish. After overnight incubation, the cells were treated with DMSO or LLL12B for 8–12 h. The cells were harvested and stained with Annexin V-APC and propidium iodide (PI) according to the manufacturer’s instructions and after staining were counted using a FACSCanto II flow cytometer (BD Biosciences, San Jose, CA, USA).

### 2.8. Colony Formation Assay

MDA-MB-231, SUM159, and 4T1 cells were seeded in a 6-well plate at a density of 1000 cells/well, treated with DMSO or LLL12B for 24 h, and then cultured in a drug-free culture medium for 7 days. The colonies were fixed with methanol for 30 min and stained with 1% crystal violet in 25% methanol for 2 h at room temperature.

### 2.9. Wound Healing Assay 

MDA-MB-231, SUM159, and 4T1 cells were seeded in a 6-well plate and cultured until fully confluent. A straight scratch crossing the monolayer was created using a 200 µL pipette tip and images of the scratched areas were taken. Following this, the cells were treated with DMSO or LLL12B and images were taken until the scratch of the DMSO-treated cells was closed and the relative migration (%) was calculated.

### 2.10. Orthotopic Mammary Fat Pad Tumor Model In Vivo

All mice were used under guidelines approved by the IACUC of University of Maryland School of Medicine. Six-week-old female athymic nude mice were purchased from The Jackson Laboratory (Bar Harbor, ME, USA). The 2.5 × 10 ^6^ MDA-MB-231 cells in 50 μL of Matrigel (BD Science, Franklin Lakes, NJ, USA) were inoculated into the 4th mammary fat pads bilaterally. After the tumor volume reached approximately 50 mm^3^, the mice were randomly divided into two groups (3 mice with 6 tumors in each group). LLL12B (2.5 mg/kg) or DMSO vehicle was administered daily by oral gavage for 28 days. Tumor size was measured by the length (L) and width (W) using a caliper ruler every 2 days. Tumor volume was calculated using 0.52 × L × W^2^. Body weight was monitored every 2 days. At the end of the experiment, the tumors were removed and weighed. Part of the tumor tissue was lysed to detect the expression of P-STAT3 (Y705), STAT3, and cyclin D1 using Western blot analysis.

### 2.11. Statistical Analysis

The statistical significance (*p* value) between the control and experimental groups was determined by Student’s unpaired *t*-test (two-group comparison) or one-way ANOVA (three-group comparison), and *p* < 0.05 was considered significant. All experiments were carried out in triplicate and the data were analyzed using GraphPad software (GraphPad Software Inc, San Diego, CA, USA).

## 3. Results

### 3.1. Knockdown of STAT3 Inhibits TNBC Cell Viability

**Figure 1:**
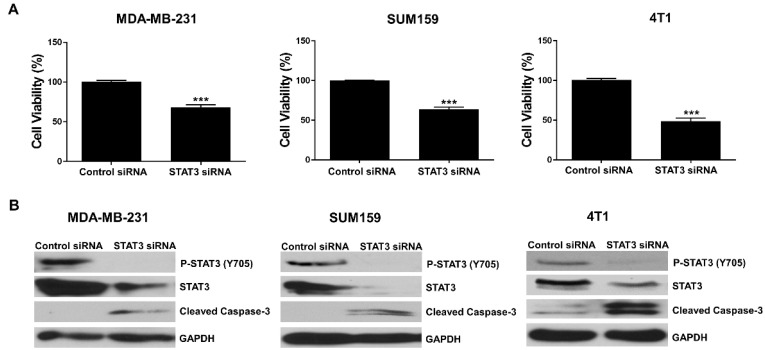
Knockdown of STAT3 inhibited TNBC cell viability. MDA-MB-231, SUM159, and 4T1 cells were transfected with control siRNA and STAT3 siRNA for 72 h and cell viability was evaluated by MTT assay (**A**). The expression levels of P-STAT3 (Y705), STAT3, and cleaved caspase-3 were analyzed by Western blot analysis (**B**). GAPDH served as a protein loading control. *** *p* < 0.001.

STAT3 is persistently activated in TNBC cells. To explore the roles of STAT3, we knocked down STAT3 expression by STAT3-specific siRNA in human TNBC cell lines MDA-MB-231 and SUM159 and murine TNBC cell line 4T1. MTT assays showed that cell viability was decreased by STAT3 knockdown in the MDA-MB-231, SUM159, and 4T1 TNBC cells compared to the cells transfected with control siRNA (Figure 1A). STAT3 knockdown efficiency was evaluated by Western blot analysis. As shown in Figure 1B, STAT3 siRNA reduced the phosphorylation of STAT3 (Y705) and total STAT3 expression, and induced the expression of the apoptosis marker, cleaved caspase-3, in the MDA-MB-231, SUM159, and 4T1 cell lines. Hence, the knockdown of STAT3 supports the role of STAT3 in maintaining cell viability in TNBC cells.

### 3.2. LLL12B Inhibits STAT3 Nuclear Translocation and Blocks IL-6-Induced STAT3 Phosphorylation

**Figure 2:**
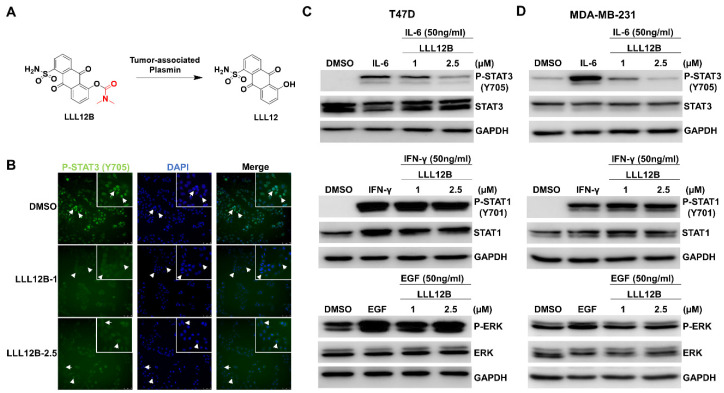
LLL12B inhibited STAT3 nuclear translocation and IL-6-induced STAT3 phosphorylation. (**A**) The chemical structures of LLL12B and LLL12. The carbamate ester bond of LLL12B is hydrolytically cleaved by the tumor-associated plasmin to release LLL12. (**B**) SUM159 cells were seeded in a 6-well plate and treated with LLL12B for 8 h. Cells were stained with Phospho-Stat3 (Tyr705) and DAPI (indicated by arrows). Green: P-STAT3 (Y705); Blue: DAPI. T47D (**C**) and MDA-MB-231 (**D**) breast cancer cells were pretreated with DMSO or LLL12B in a serum-free medium for 2 h and stimulated with 50 ng/mL of IL-6, IFN-γ, or EGF for additional 30 min. Cells were collected and analyzed by Western blot analysis. GAPDH served as a protein loading control.

Carbamate esters are one of the prodrug types which are developed to improve the pharmacokinetic properties of drugs [29,30]. A carbamate prodrug of STAT3 inhibitor LLL12, named LLL12B, which is activated by the tumor-associated plasmin, was designed and tested in this study (Figure 2A). The nuclear translocation of phosphorylated STAT3 (Y705) is required for regulating the transcription of STAT3 target genes. Thus, we examined the cellular localization of P-STAT3 (Y705) in SUM159 cells treated with LLL12B using immunofluorescence staining. In the DMSO-treated SUM159 cells, P-STAT3 (Y705) was mainly present in the nucleus. However, the distribution of P-STAT3 (Y705) in the nucleus was blocked by the LLL12B treatment (Figure 2B), supporting that the STAT3 inhibitor LLL12B was activated in the SUM159 cells. We have shown that LLL12B inhibited IL-6-induced STAT3 phosphorylation in human medulloblastoma cells [25,31]. To further confirm the selectivity of LLL12B in breast cancer cells, we tested its effects on the phosphorylation of STAT3, STAT1, or ERK following the stimulation with IL-6, IFN-γ, or EGF in the T47D breast cancer cells, which express lower basal levels of P-STAT3 (Figure 2C), and in the MDA-MB-231 TNBC cells (Figure 2D). As shown by Western blot analysis, IL-6 induced the phosphorylation of STAT3 (Y705) and the induction of P-STAT3 was inhibited by LLL12B in both the T47D and MDA-MB-231 cell lines. However, LLL12B did not inhibit the induction of P-STAT1 (Y701), which is stimulated by IFN-γ in the T47D and MDA-MB-231 cell lines, or the induction of P-ERK, which is stimulated by EGF in the T47D cells. There was a slight induction of P-ERK stimulated by EGF in the MDA-MB-231 cells, and LLL12B had little effect on the inhibition of P-ERK. Collectively, these results confirm that LLL12B selectively inhibits the IL-6/STAT3 signaling pathway.

### 3.3. LLL12B Inhibits STAT3 Activation and Induces TNBC Cell Apoptosis

**Figure 3:**
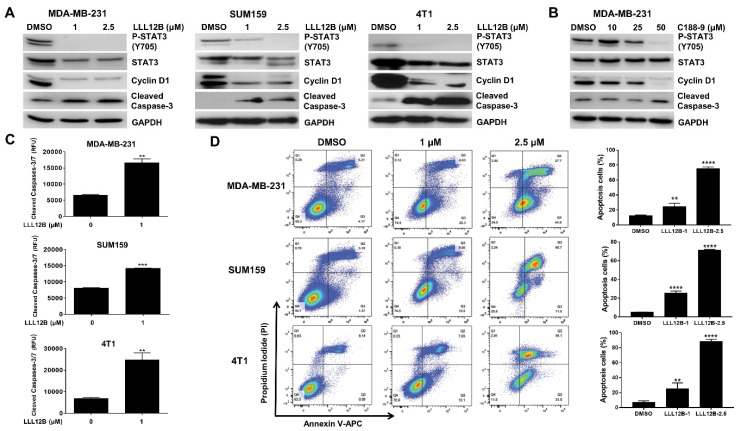
LLL12B inhibited STAT3 phosphorylation and induced apoptosis in TNBC cells. (**A**) MDA-MB-231, SUM159, and 4T1 cell lines were treated with DMSO or LLL12B overnight and the expression levels of P-STAT3 (Y705), STAT3, cyclin D1, and cleaved caspase-3 were analyzed by Western blot analysis. GAPDH served as a protein loading control. (**B**) MDA-MB-231 cells were treated with DMSO or C188-9 overnight and the expression levels of P-STAT3 (Y705), STAT3, cyclin D1, and cleaved caspase-3 were analyzed by Western blot analysis. (**C**) MDA-MB-231, SUM159, and 4T1 cells were seeded in a 96-well plate and treated with DMSO or LLL12B for 5 h and a Caspase-3/7 Fluorescence Assay Kit was used to detect the activation of caspase-3/7. (**D**) MDA-MB-231, SUM159, and 4T1 cell lines were treated with DMSO or LLL12B for 8–12 h, and flow cytometry was performed to analyze annexin V and PI staining. The quantification of apoptotic cells was shown in the right panel. ** *p* < 0.01, *** *p* < 0.001 and **** *p* < 0.0001.

To explore the activity of LLL12B in the TNBC cells, we treated the MDA-MB-231, SUM159, and 4T1 cell lines with LLL12B and the vehicle control DMSO. As shown in Figure 3A, Western blot analysis demonstrated that the phosphorylation of STAT3 and its downstream target cyclin D1 were decreased in the LLL12B-treated cells compared to the DMSO-treated cells. Additionally, the expression of cleaved caspase-3 was enhanced in the LLL12B-treated cells. As a comparison, the MDA-MB-231 cells were treated with the oral STAT3 inhibitor C188-9, also named TTI-101, which is currently in phase I clinical study for advanced solid tumors [32]. As shown in Figure 3A, 1 µM of LLL12B effectively inhibited P-STAT3, and the STAT3 downstream target cyclin D1, as well as induced cleaved caspase-3 in the MDA-MB-231 cells, while 25 µM and 50 µM of C188-9 were needed to inhibit P-STAT3 and cyclin D1 (Figure 3B). In conjunction with Western blot analysis, the caspase-3/7 activity assay demonstrated that the LLL12B treatment induced the activation of caspase-3/7 in the MDA-MB-231, SUM159, and 4T1 cell lines (Figure 3C). To further confirm cell apoptosis, we performed annexin V and PI staining using flow cytometry. As shown in Figure 3D, the percentage of apoptotic cells was significantly increased in the LLL12B-treated cells compared to the DMSO-treated cells. Overall, these results indicate that LLL12B is a potent STAT3 inhibitor and induces apoptosis in TNBC cells.

### 3.4. LLL12B Inhibits Colony Formation in the TNBC Cells

**Figure 4:**
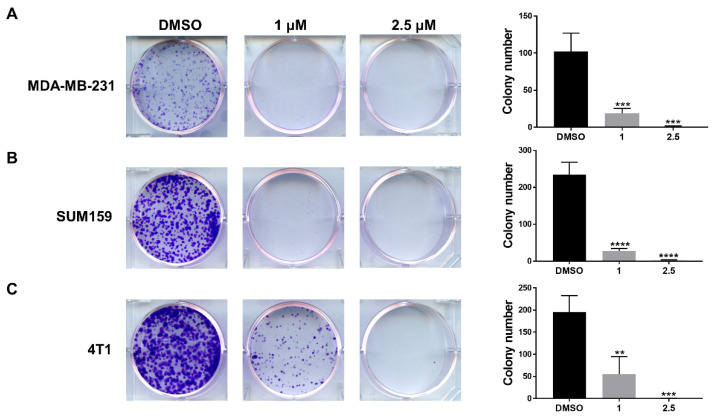
LLL12B suppressed colony formation of TNBC cells. MDA-MB-231 (**A**), SUM159 (**B**), and 4T1 (**C**) cells were seeded in a 6-well plate and treated with DMSO or LLL12B for 24 h and recovered in a drug-free culture medium for 7 days. Representative images of colony formation are shown in the left panel and the colony number was quantified and is shown in the right panel. ** *p* < 0.01, *** *p* < 0.001 and **** *p* < 0.0001.

To investigate the long-term effect of LLL12B on the expansion of TNBC cells, the colony formation assay was performed. MDA-MB-231, SUM159, and 4T1 cells were treated with LLL12B or DMSO for 24 h and recovered in a drug-free culture medium for 7 days. As shown in Figure 4, the LLL12B treatment showed a significant reduction in colony numbers compared to the DMSO treatment.

### 3.5. LLL12B Inhibits Cell Migration in the TNBC Cells

**Figure 5:**
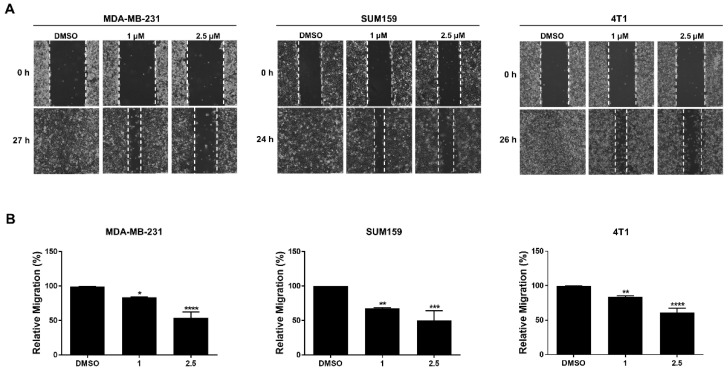
LLL12B inhibited migration in TNBC cells. (**A**) Wound healing assay was performed to detect the cell migration in MDA-MB-231, SUM159, and 4T1 cell lines with LLL12B treatment. Cell images show the initial and final scratches. (**B**) Quantification analysis of the wound area is shown. * *p* < 0.05, ** *p* < 0.01, *** *p* < 0.001 and **** *p* < 0.0001.

The migration of cancer cells is relevant to their ability to metastasize; therefore, we determined the inhibitory effect of LLL12B on cell migration by performing a wound healing assay in the MDA-MB-231, SUM159, and 4T1 cell lines. Notably, LLL12B treatment, compared to DMSO treatment, effectively inhibited wound closure in the MDA-MB-231, SUM159, and 4T1 cell lines (Figure 5). Therefore, these results provide experimental evidence that LLL12B is capable of inhibiting the migration of TNBC cells.

### 3.6. LLL12B Suppresses Tumor Growth in the MDA-MB-231 Orthotopic Tumor Model In Vivo

**Figure 6:**
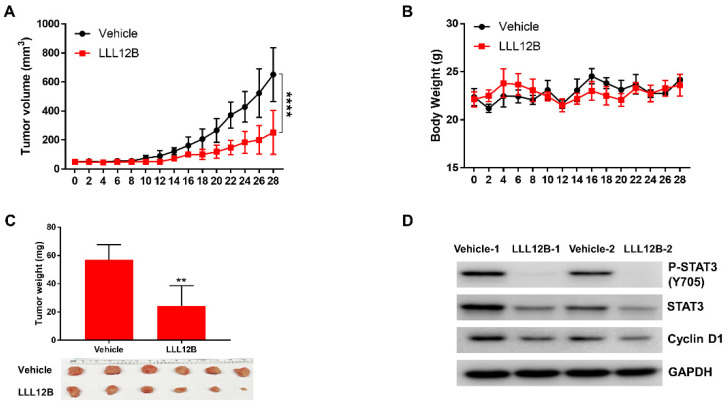
LLL12B suppressed MDA-MB-231 tumor growth in vivo. MDA-MB-231 cells were inoculated into the 4th mammary fat pad, and vehicle control or LLL12B (2.5 mg/kg) was administered orally every day for 28 days. (**A**) Tumor volumes were measured every 2 days. (**B**) Body weights were measured every 2 days. (**C**) Tumors were removed after 28 days of treatment and tumor weights were measured. Tumor samples are shown in the bottom panel. (**D**) The expression levels of P-STAT3 (Y705), STAT3, and cyclin D1 in the vehicle- and LLL12B-treated tumor samples were analyzed by Western blot analysis. GAPDH served as a protein loading control. ** *p* < 0.01, **** *p* < 0.001.

Female nude mice were implanted with the MDA-MB-231 cells to establish an orthotopic tumor model to further evaluate LLL12B efficacy in vivo. MDA-MB-231 cells were inoculated into the fourth mammary fat pad and tumors were allowed to develop until the volume reached approximately 50 mm^3^. Vehicle control or LLL12B (2.5 mg/kg) was administered once a day by oral gavage for 28 days. Compared to the vehicle-treated group, the tumor volume in the LLL12B-treated group was significantly reduced (Figure 6A). In addition, the LLL12B-treated group showed reduced tumor weight (Figure 6C). There was no obvious reduction in body weight observed in the LLL12B-treated group compared to the vehicle-treated group (Figure 6B) suggesting little acute toxicity. As expected, Western blot analysis of the tumor tissues showed that LLL12B suppressed the phosphorylation of STAT3 (Y705) and cyclin D1 expression (Figure 6D). Collectively, these results indicate that LLL12B, as a single agent, can suppress MDA-MB-231 tumor growth in vivo.

## 4. Discussion

Given the increasing evidence supporting STAT3 as a pivotal target for cancer therapy, novel inhibitors targeting STAT3 will have a considerable inhibitory impact on cancers that harbor constitutively activated STAT3, such as TNBC. In this study, we evaluated the antitumor activity of LLL12B in TNBC cells and provided experimental evidence for a potential therapeutic strategy for TNBC treatment.

STAT3 is one of the STAT family proteins including STAT1, STAT2, STAT3, STAT4, STAT5A, STAT5B, and STAT6. They share a homologous domain structure composed of an amino-terminal domain, a coiled-coil domain, a DNA-binding domain, an α-helical linker domain, an SRC-homology 2 (SH2) domain, and a carboxy-terminal transactivation domain [33,34,35]. These similarities make it more challenging to target STAT3 specifically. In particular, both STAT1 and STAT3 are involved in tumorigenesis, but play opposite roles [36]. The canonical STAT3 signaling pathway is activated by the binding of cytokines or growth factors to their corresponding receptors [37]. Among them, IL-6 is critical for STAT3 activation in human breast cancer [13,38]. In the present study, we found LLL12B selectively inhibited IL-6-mediated STAT3 activation but had little effect on IFN-γ-mediated STAT1 activation and EGF-mediated ERK activation (Figure 2), supporting LLL12B as a selective STAT3 inhibitor.

STAT3 is a valuable target for anticancer drug development. Various strategies targeting STAT3 directly or upstream kinases indirectly have been tested, including peptides, small molecules, oligonucleotides, and natural compounds [22,39,40,41]. Because of the off-target toxicities of STAT3 upstream kinase inhibitors, lacking the stability of peptides and lacking the effective delivery of oligonucleotides, small molecular inhibitors targeting STAT3 directly continue to be the most common approach. To date, the majority of small-molecule STAT3 inhibitors are designed to target the SH2 domain which is essential for STAT3 dimerization. A number of small-molecule inhibitors of STAT3 targeting the SH2 domain have been reported and shown antitumor activity, such as Stattic [42], S3I-201 [43], C188-9 [32], and Bt354 [44]. We have developed several STAT3 inhibitors targeting the SH2 domain using computer-aided drug design, including LLL12, LLY17, and LLL12B [23,24,25,27,28]. LLL12B is a prodrug of LLL12 with superior in vivo pharmacokinetic properties compared to LLL12 [25,28]. It has been shown that LLL12B induced apoptosis, suppressed tumor growth, and exhibited synergistic activity in combination with cisplatin or irradiation in medulloblastoma cells [25,31]. In addition, LLL12B sensitized ovarian cancer cells to paclitaxel and cisplatin [45]. In this study, we evaluated the inhibitory effects of LLL12B on TNBC cells in vitro and in vivo. Our results demonstrated that LLL12B induced apoptosis and suppressed colony formation and migration in TNBC cells (Figure 3, Figure 4 and Figure 5). Our results also showed that LLL12B downregulated the STAT3 downstream gene cyclin D1, which is involved in cancer cell proliferation (Figure 3). Furthermore, LLL12B exhibited antitumor activity in the MDA-MB-231 orthotopic tumor model in vivo (Figure 6). In addition, LLL12B was more potent than the oral STAT3 inhibitor C188-9 in the inhibition of STAT3 phosphorylation and cyclin D1, as well as the induction of apoptosis (Figure 3). Since the STAT3 inhibitor C188-9 is currently in phase I clinical study for advanced solid tumors [32], the superior activity of LLL12B supports the potential for it to possibly be used in clinical studies in the future.

In summary, our results demonstrate that targeting STAT3 with LLL12B induces apoptosis, inhibits colony formation and cell migration in vitro, and suppresses tumor growth of TNBC cells in vivo. These results support that LLL12B is a potential therapeutic agent either to be used as monotherapy or in combination with another therapeutic agent for TNBC treatment.

## Data Availability

The data presented in this study are available in this article.
